# Association between tocilizumab and emerging multidrug-resistant organisms in critically ill patients with COVID-19: A multicenter, retrospective cohort study

**DOI:** 10.1186/s12879-021-06813-1

**Published:** 2021-11-01

**Authors:** Ohoud Aljuhani, Khalid Al Sulaiman, Adel Alshabasy, Khalid Eljaaly, Abdulrahman I. Al Shaya, Haytham Noureldeen, Mohammed Aboudeif, Bodoor Al Dosari, Amina Alkhalaf, Ghazwa B. Korayem, Muneera M. Aleissa, Hisham A. Badreldin, Shmeylan Al Harbi, Abdullah Alhammad, Ramesh Vishwakarma

**Affiliations:** 1 Department of Pharmacy Practice, Faculty of Pharmacy, King Abdulaziz University, P. O. Box 80260, Jeddah, 21589 Saudi Arabia; 2 Pharmaceutical Care Department, King Abdulaziz Medical City, Riyadh, Saudi Arabia; 3 Department of Anesthesia and Intensive Care, King Abdulaziz University, Jeddah, Saudi Arabia; 4 College of Pharmacy, King Saud Bin Abdulaziz University for Health Sciences, Riyadh, Saudi Arabia; 5 King Abdullah International Medical Research Center, Riyadh, Saudi Arabia; 6 Pharmaceutical Care Department, King Abdulaziz University Hospital, Jeddah, Saudi Arabia; 7 College of Pharmacy, Princess Nourah Bint Abdulrahman University, Riyadh, Saudi Arabia; 8 Department of Clinical Pharmacy, College of Pharmacy, King Saud University, Riyadh, Saudi Arabia; 9 College of Pharmacy, University of Arizona, Tucson, AZ USA; 10 Brigham and Women’s Hospital, Boston, MA USA; 11 Department of Anesthesia and Intensive Care, Ain Shams University, Cairo, Egypt

**Keywords:** COVID-19, SARS-Cov-2, Tocilizumab, Secondary infection, CRE, Critically ill, Intensive care units (ICUs), Immunomodulatory drugs

## Abstract

**Background:**

Tocilizumab is an IgG1 class recombinant humanized monoclonal antibody that directly inhibits the IL-6 receptor. Several randomized clinical trials have evaluated its safety and efficacy in patients with coronavirus disease 2019 (COVID-19), and these studies demonstrate conflicting results. Our study aimed to determine the association between tocilizumab treatment and microbial isolation and emergence of multidrug-resistant bacteria in critically ill patients with COVID-19.

**Methods:**

A multicenter retrospective cohort study was conducted at two tertiary government hospitals in Saudi Arabia. All critically ill patients admitted to intensive care units with a positive COVID-19 PCR test between March 1 and December 31, 2020, who met study criteria were included. Patients who received tocilizumab were compared to those who did not receive it.

**Results:**

A total of 738 patients who met our inclusion criteria were included in the analysis. Of these, 262 (35.5%) received tocilizumab, and 476 (64.5%) were included in the control group. Patients who received tocilizumab had higher odds for microbial isolation (OR 1.34; 95% CI 0.91–1.94, p = 0.13); however, the difference was not statistically significant. Development of resistant organisms (OR 1.00; 95% CI 0.51–1.98, p = 0.99) or detection of carbapenem-resistant Enterobacteriaceae (CRE) (OR 0.67; 95% CI 0.29–1.54, p = 0.34) was not statistically significant between the two groups.

**Conclusions:**

Tocilizumab use in critically ill patients with COVID-19 is not associated with higher microbial isolation, the emergence of resistant organisms, or the detection of CRE organisms.

**Supplementary Information:**

The online version contains supplementary material available at 10.1186/s12879-021-06813-1.

## Background

Since the emergence of the novel coronavirus (SARS-CoV-2) in December 2019 in Wuhan, China [1], the world has been dealing with a new highly contagious disease. As of August 15, 2021, the novel coronavirus disease 2019 (COVID-19) has infected over 206 million people worldwide, with crude mortality exceeding 4.3 million people [1]. Patients hospitalized with COVID-19 often present with pneumonia due to an excessive host immune response causing acute respiratory distress syndrome [2]. Respiratory distress is associated with increased intensive care unit (ICU) admission and mortality [3].

It has been hypothesized that the occurrence of severe respiratory distress in critically ill patients is attributed to a state known as cytokine release syndrome (CRS), in which the body produces pro-inflammatory cytokines (TNF-α, IL-1β, IL-2, and IL-6) and chemokines (IL-8) [4]. Recent studies have shown increased cytokine levels, specifically interleukin 6 (IL-6), in critically ill patients with COVID-19 [3]. This suggests that elevated IL-6 levels may be a marker of poor prognosis in patients with COVID-19 [4]. Thus, the use of therapeutic agents targeting IL-6, such as tocilizumab, has been studied in hospitalized patients with COVID-19 and showed mortality benefits and associated with clinical improvement [5–8].

Tocilizumab is an IgG1 class recombinant humanized monoclonal antibody that directly inhibits the IL-6 receptor [9]. Several randomized clinical trials (RCTs) have evaluated the safety and efficacy of tocilizumab in patients with COVID-19 and demonstrated conflicting results [5–7, 10, 11]. One RCT, including 389 non-mechanically ventilated (MV) patients with COVID-19, reported a decrease in the likelihood of progression to MV or death in the tocilizumab group compared to standard therapy [6]. In large RCTs conducted on hospitalized patients with COVID-19 experiencing progressive symptoms, tocilizumab use was associated with mortality benefits [6, 7, 10].

Although tocilizumab has an anti-inflammatory effect, this effect can lead to the impairment of host response, predisposing the patients to serious secondary bacterial, viral, and fungal infections, most commonly upper respiratory tract infections [12]. Further, critically ill patients are more prone to additional risks of developing secondary infections and resistant microorganisms due to the increased use of antibiotics and circulatory support devices. Moreover, reports of secondary bacterial infections in patients with COVID-19 vary from 4 to 25%, reaching up to 50% [13, 14]. The addition of tocilizumab and systemic steroids can severely diminish the patient’s immune system, increasing the risk of exposure to resistant organisms. Thus, we sought to evaluate the association between tocilizumab treatment with microbial isolation and the emergence of multidrug-resistant bacteria among critically ill patients with COVID-19.

## Methods

### Study design and participants

**Fig. 1:**
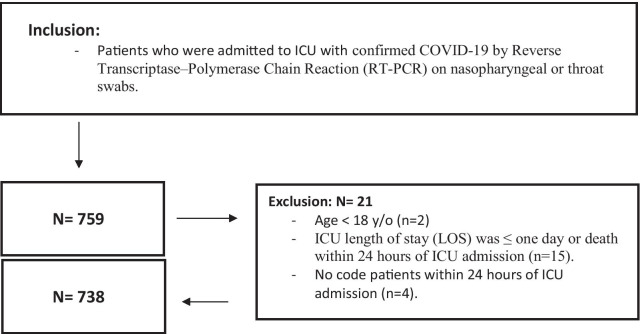
Flowchart

This was a multicenter retrospective cohort study that evaluated the risk of developing multidrug-resistant organisms (MDRO) in adult critically ill patients with COVID-19 (aged ≥ 18 years) who received tocilizumab between March 1 and December 31, 2020. COVID-19 was diagnosed using reverse transcriptase-polymerase chain reaction (RT-PCR) obtained from nasopharyngeal or throat swabs. Patients were excluded if the ICU length of stay (LOS) was less than one day or if they had a “Do-Not-Resuscitate” code status within 24 h of ICU admission (Fig. 1). Eligible patients were classified into two groups based on tocilizumab use during the ICU stay.

### Setting

This study was conducted in two tertiary governmental hospitals. The primary site was King Abdulaziz University Hospital, Jeddah. This study was approved by the Biomedical Ethics Research Committee at the Faculty of Medicine at King Abdulaziz University (Reference number 15-21). The second site was King Abdulaziz Medical City, Riyadh, and the study was approved by King Abdullah International Medical Research Center in February 2021 (Reference number NRC21/025/01).

During the study period, the ICUs of the participating hospitals had no carbapenem-resistant *Enterobacteriaceae* (CRE) outbreaks. All participating institutions were mandated to follow standard precautions for patients with confirmed or suspected infections, such as multidrug-resistant (MDR), extensive drug-resistant (XDR), and pan drug-resistant (PDR) infections. All sites followed the Saudi Center for Disease Prevention and Control guidelines, including patients’ isolation and universal masking of all healthcare workers, patients, and visitors [15, 16].

### Data collection

The following details were collected from the electronic health record: demographic data, comorbidities, vital signs, laboratory tests, severity scores (i.e., acute physiology and chronic health evaluation II [APACHE II], sequential organ failure assessment [SOFA] scores), Glasgow coma score (GCS), acute kidney injury, the need for MV, and MV parameters (PaO_2_/FiO_2_ (P/F) ratio, FiO_2_ requirement) within 24 h of ICU admission (Additional file 1: Table S1). Additionally, renal profile, liver function tests, coagulation profile (INR, aPTT, fibrinogen), and inflammatory markers (CRP, procalcitonin) within 24 h of ICU admission were collected. Moreover, culture information, including the presence of resistant organisms, was collected during the ICU stay. Tocilizumab and systemic corticosteroid use were recorded for eligible patients. All patients were followed up until they were discharged from the hospital or died during the hospital stay, whichever occurred first.

### Outcomes

The primary outcome was to estimate the prevalence of microbial isolation in critically ill patients with COVID-19 who received tocilizumab. The secondary outcomes of interest included resistant organisms, CRE emergence, hospital LOS, ICU LOS, and MV duration.

Bacteria and fungi were identified in the blood, urine, wound, drainage, cerebrospinal fluid, and respiratory specimens. Microbial isolates defined as sputum or endotracheal aspiration showed growth of ≥ 100,000 CFU/mL. Further, bronchoalveolar lavage (BAL) showed growth of ≥ 10,000 CFU of single organism/mL for protected specimen brushes (PSBs) and ≥ 100,000 CFU of single organism/mL for BAL fluid. Additionally, urine cultures were considered significant if they showed growth of ≥ 100,000 CFU/mL of no more than two species of microorganisms [16]. Cultures were excluded if the laboratory reported them as contaminants.

### Definition (s)

- Multidrug-resistant organisms (MDRO) are not susceptible to at least one agent in three or more classes of antibiotics.
- Extensive drug-resistant (XDR) organisms are not susceptible to at least one agent in all, but two or fewer antimicrobial classes remain susceptible.
- Pan drug-resistant (PDR) organisms are not susceptible to all agents in all antimicrobial classes.
- Susceptibility of gram-negative bacteria was created using documents and breakpoints based on the Clinical Laboratory Standards Institute (CLSI) [17, 18].
- Carbapenem-resistant *Enterobacteriaceae* (CRE) have been defined as carbapenem-nonsusceptible and extended-spectrum cephalosporin-resistant *Escherichia coli*, *Enterobacter cloacaecomplex*, *Klebsiella aerogenes, Klebsiella pneumoniae*, or *Klebsiella oxytoca*, these may be secondary to metallo-betalactamases, zinc metalloenzymes (e.g., New Dehli Metallo (NDM),VIM-1, IMP-1), ampC beta-lactamase, and oxacillinases (e.g., OXA-23-like, OXA-48, OXA-58-like OXA-48) [19].

### Data management and statistical analysis

Categorical data were expressed as numbers and percentages. Continuous variables were expressed as mean and standard deviation (SD) if they were normally distributed, or median and first quartile (Q1) and third quartile (Q3) if they were not normally distributed.

Categorical variables were analyzed using the Chi-square or Fisher exact test, and continuous variables were analyzed using Student’s t-test or the Mann–Whitney U test, as appropriate. Multivariable logistic regression was conducted to evaluate the microbial isolation, resistant organisms, CRE emergence after adjusting for possible co-founders including the following: patient comorbidities (i.e., diabetes mellitus, chronic kidney disease (CKD) on dialysis), history of hospitalization or invasive procedure (surgery) within 1 year, history of antibiotic exposure in the last 3 months, and systemic corticosteroid use during ICU and ICU LOS. To reduce the risk of bias in the regression model, we used the causal directed acyclic graph approach to ensure the inclusion of several confounders in the regression model that have been associated with an increased risk of developing MDR strains [20–23]. For other outcomes (MV, ICU, and hospital LOS), multivariable generalized linear regression was used to assess the relationship between tocilizumab use and the different outcomes considered in this study after adjusting for the patients’ age, SOFA score, PaO_2_/FiO_2_ ratio baseline, and systemic corticosteroids during ICU. The Odds ratios (ORs) and estimates with 95% confidence intervals (CIs) were reported for the associations, as appropriate.

We assessed model fit using the Hosmer–Lemeshow goodness-of-fit test. No imputation was made for missing data, as the cohort of patients in our study was not derived from random selection. Statistical significance was set at p < 0.05. All statistical analyses were performed using SAS version 9.4 for all statistical analyses.

## Results

A total of 738 patients met our inclusion criteria and were included in the final analysis. Of these, 262 (35.5%) received tocilizumab, and 476 (64.5%) who did not receive tocilizumab were included in the control group. The median (Q1, Q3) dose of tocilizumab administered per day was 400 mg (400 mg, 600 mg). Approximately 39% of the patients received tocilizumab early within 24 h of ICU admission.

### Participants’ clinical characteristics

For all patients, the mean (SD) age was 61 years (14.7), and most patients were male (72.1%). We observed several notable differences between the two groups: patients who received tocilizumab were younger compared to those in the control group, had significantly higher C-reactive protein (CRP) levels, had significantly lower PaO_2_/FiO_2_ ratio, and were more likely to receive systemic corticosteroids in the ICU (93.4% vs. 84.9%). Conversely, patients who did not receive tocilizumab were more likely to have a history of hospitalization or surgery within a year (20.5% vs. 7.4%). Additionally, the prevalence of diabetes, CKD, and ischemic heart disease were significantly higher in the control group (Additional file 1: Table S1).

### Primary outcomes

Overall, there were statistically insignificant differences between the two groups in terms of the prevalence  of microbial isolation (44.9% vs. 50.5%, p = 0.14). Moreover, after adjusting for possible confounders, receiving tocilizumab was not associated with an increased odds of microbial isolation (OR, 1.34; 95% CI 0.91–1.94, p = 0.13) (Table 1).

**Table 1:** Regression analysis for the outcomes

Outcomes	Crude analysis		p-value^^^^	Odds ratio (OR) (95%CI)	p-value for adjusted analysis^$^**
	Control group	Tocilizumab group			
Secondary infection (Bacterial/fungal), n (%)	214/476 (44.9)	132/261 (50.5)	0.14	1.34 (0.91, 1.94)	0.13
Resistant organisms (i.e. MDR, EDR, PDR), n (%)	82/141(58.1)	54/84 (64.2)	0.36	1.00 (0.51, 1.98)	0.99
CRE detection, n (%)	21/476 (4.4)	10/262 (3.8)	0.69	0.67 (0.29, 1.54)	0.34

^^^^Chi-square test is used to calculate the p-value

^$^**Multivariable Logistic regression is used after adjusting for patient’s comorbidities (i.e. DM, CKD on dialysis), history of hospitalization or invasive procedure (Surgery) within 1 year, history of antibiotics exposure in the last 3 months, systemic corticosteroids during ICU and ICU LOS to calculate odds ratio and p-value

### Secondary outcomes

In the crude analysis, there were statistically insignificant differences in terms of the emergence of resistant organisms (58.1% vs. 64.2%, p = 0.36) and CRE detection (4.4% vs. 3.8%, p = 0.69) (Table 1). Results from the multivariable logistic regression analysis demonstrated similar odds of emergence of resistant organisms (OR 1.00; 95% CI 0.51–1.98, p = 0.99) and CRE detection (OR 0.67; 95% CI 0.29–1.54, p = 0.34) (Table 1).

The hospital LOS (18 days vs. 16 days, p = 0.04) and ICU LOS (9 days vs. 8 days, p = 0.01) were significantly higher in the tocilizumab group than in the control group. However, they were statistically insignificant after adjusting for possible confounders (age, baseline SOFA score, PaO_2_/FiO_2_ ratio, and systemic corticosteroid use) using negative binomial regression analysis (Table 2).

**Table 2:** Regression analysis for MV duration and LOS

Outcomes	Crude analysis		p-value	Beta coefficient (estimates) (95% CI)	p-value^$^*
	Control group	Tocilizumab group			
MV duration during ICU stay days, median (Q1, Q3)^&#^	9.0 (4.0, 17.0)	10.0 (4.0, 18.0)	0.46	0.06 (− 0.11, 0.22)	0.49
ICU length of stay days, median (Q1, Q3)^&^	8.0 (5.0, 13.0)	9.0 (6.0, 15.0)	0.01	0.12 (− 0.05, 0.29)	0.16
Hospital length of stay days, median (Q1, Q3)^&^	16.0 (11.0, 26.0)	18.0 (13.0, 27.5)	0.04	0.15 (− 0.00, 0.29)	0.05

^$*^Multivariable negative binomial regression is used after adjusting for patient’s age, SOFA score, PaO_2_/FiO_2_ ratio baseline and systemic corticosteroids during ICU stay to calculate estimates and p-value

^&^^**#**^Denominator is patients who have respiratory failure requiring MV during ICU stay

^&^Denominator is patients who survived

The follow-up inflammatory markers were statistically significant among the groups. d-dimer and iron levels were higher in the tocilizumab group, but fibrinogen was lower in the tocilizumab group. CRP and procalcitonin levels were similar among the groups, as shown in Table 3.

**Table 3:** Follow-up for inflammatory markers

Outcomes	Crude analysis		p-value^^^	Beta coefficient (estimates) (95% CI)	p-value^$^*
	Control group	Tocilizumab group			
Serum iron level follow-up, Median (Q1, Q3)	5.88 (3.70, 10.23)	8.91 (3.98, 13.66)	0.004	0.31 (0.15, 0.47)	0.0002
D-dimer level follow-up (mg/l), Median (Q1, Q3)	3.24 (1.13, 8.51)	4.01 (1.53,14.74)	0.006	2.81 (2.39, 3.24)	< 0.0001
Fibrinogen level follow-up (gm/l), Median (Q1, Q3)	6.29 (4.59, 7.85)	5.43 (3.60, 7.30)	0.0003	− 0.18 (− 0.28, − 0.08)	0.0002
CRP level follow-up (mg/l), Median (Q1, Q3)	164.0 (84.0, 261.0)	187.0 (93.0, 302.0)	0.14	0.11 (− 0.05, 0.27)	0.18
Procalcitonin level follow-up (ng/ml), Median (Q1, Q3)	0.46 (0.13, 1.73)	0.55 (0.15, 3.32)	0.09	− 0.37 (− 1.77, 1.03)	0.60

^^^Wilcoxon rank sum test is used to calculate the p-value

^$^* Multivariate Logistic regression is used after adjusting for patient’s age, SOFA score, PaO_2_/FiO_2_ ratio baseline and systemic corticosteroids during ICU stay to calculate estimates and p-value

## Discussion

In our retrospective cohort study of critically ill patients with COVID-19, we observed that patients who received tocilizumab neither had a significantly higher risk of acquiring resistant bacteria such as MDR, XDR, and PDR nor had a higher risk of CRE. Moreover, the tocilizumab group had similar MV duration, ICU LOS, and hospital LOS to those who did not receive tocilizumab.

Our primary outcome of no difference in microbial isolation rate was observed after adjusting for possible co-founders. This finding is similar to the results of previous studies that conducted adjusted matched analyses for tocilizumab and controls and showed no difference between the groups [24, 25]. This was also demonstrated in another multicenter cohort study of 4485 adults with COVID-19 admitted to ICUs and a recent meta-analysis that found no differences in secondary infection rate between tocilizumab and the control group [5, 26, 27]. However, several published trials have found lower rates of secondary infections with tocilizumab [10, 11, 28]. The differences in outcomes in these studies could be attributed to the unadjusted analysis in some studies and the lack of a standardized definition for secondary infections.

Conversely, one study found higher rates of secondary infections with tocilizumab use. However, this was limited by the study design and the short follow-up period to confirm the association between tocilizumab and the higher rate of secondary infections [29]. Another recently published retrospective cohort study conducted on critically ill patients with COVID-19 found that multiple doses of tocilizumab were associated with higher odds of pneumonia than a single dose [30].

In our multivariable logistic regression analysis, we demonstrated similar odds of the emergence of resistant organisms. Although earlier observational studies have suggested an association between tocilizumab use and the emergence of secondary infections [29], in our report, this association could not be made. Additionally, a study evaluated the risk factors for MDR bacteria in critically ill patients with COVID-19 and concluded no independent association between tocilizumab use and isolation of MDR gram-negative bacteria [31]. Notably, several studies that reported higher rates of infections after tocilizumab use were limited by not adjusting for steroid use—an important cofounder that we considered [29, 32]. Further, these studies vary in terms of the follow-up periods after tocilizumab use, reaching up to 8 weeks in one study showing that a longer follow-up period was linked with higher infection rates [12].

There was no significant difference in multivariable logistic regression analysis between the two groups regarding CRE detection (p = 0.34). Our results contradict those of a prospective observational study conducted to assess the predictors of superinfections in patients with COVID-19 [32]. This study showed that CRE caused 52 episodes of MDR infections and reported a significant difference (p = 0.001) between IL6 users and non-users in terms of superinfection rate. This association was not apparent in our study. However, Falcone et al. concluded that the use of immunomodulators (tocilizumab or baricitinib) was considered as a predictor independently associated with the development of superinfections [33].

In this study, tocilizumab use was not associated with any statistically significant difference in ICU and hospital LOS. Conversely, the recently published RECOVERY trial showed that tocilizumab use was associated with a greater probability of discharge from hospital within 28 days (57% vs. 50%; rate ratio 1.22, 1.12–1.33, p < 0.0001 [7]. Additionally, another trial found that until patients were discharged from the hospital, the median time was 20 days in the tocilizumab group and 28 days in the placebo group [5]. They report shorter ICU LOS—the median duration of ICU stay was 9.8 days in the tocilizumab group and 15.5 days in the placebo group, for a difference of 5.8 days [5].

Among patients who received tocilizumab, we found differences in the levels of follow-up inflammatory markers compared with the control group. d-dimer levels were higher in the tocilizumab group than in the placebo group. In line with these results, Rossotti et al. found that d-dimer levels rose by day 5 and then decreased but did not return to baseline values [34]. This finding is consistent with previous observational studies [35, 36], where CRP and IL-6 levels improved; however, d-dimer levels increased significantly [32]. Nevertheless, a recent single-center prospective study aimed to evaluate the effect of high-dose tocilizumab on laboratory biomarkers found that CRP, IL-6, and ferritin levels were significantly lower among survivors (*p* < 0.01) [37].

Our study has some limitations that need to be addressed. Although we conducted a multivariable analysis for possible confounders, the retrospective and observational nature of the study may leave some risk for residual confounders. Furthermore, the time between tocilizumab initiation and the impact of multiple doses of tocilizumab on the emergence of microbial isolation and MDR were not assessed. Additionally, the short follow-up period may limit the ability to capture the development of infection after hospital discharge. We did not conduct a sample size calculation; therefore, this study may be underpowered to detect statistically significant differences. Further studies are needed to characterize the timeline of developing secondary infections in critically ill patients with COVID-19 treated with immunomodulators.

## Conclusion

Overall, our study demonstrated that tocilizumab use in critically ill patients with COVID-19 was not associated with higher microbial isolation, emergence of resistant organisms, or the detection of CRE organisms. Further randomized clinical and interventional studies are required to confirm our findings.

## Supplementary Information

**Additional file 1: Table S1.** Summary of demography, baseline characteristics, and co-existing illness.

## Data Availability

The datasets used and/or analyzed during the current study are available from the corresponding author on reasonable request.

## Abbreviations

ICUs: Intensive care units
COVID-19: Coronavirus disease 2019
RCTs: Randomized clinical trials
MV: Mechanical ventilation
LOS: Length of stay
CRE: Carbapenem-resistant *enterobacterales*
CRS: Cytokine release syndrome
IL-6: Interleukin 6
MDRO: Multi-drug resistance organisms
RT-PCR: Reverse transcriptase-polymerase chain reaction
XDR: Extensive drug-resistant
PDR: Pan drug-resistant
APACHE II: Acute physiology and chronic health evaluation II
SOFA: Sequential organ failure assessment
GCS: Glasgow coma score
AKI: Acute kidney injury
LFTs: Liver function tests
CSF: Cerebrospinal fluid
BAL: Bronchoalveolar lavage
CLSI: Clinical laboratory standards institute
DM: Diabetes mellitus
CKD: Chronic kidney disease
CRP: C-reactive protein
