# Supplementary material for: Association between tocilizumab and emerging multidrug-resistant organisms in critically ill patients with COVID-19: A multicenter, retrospective cohort study
Source: BMC Infect Dis. 2021 Nov 1;21:1127. doi: 10.1186/s12879-021-06813-1 (PMC8559694; doi:10.1186/s12879-021-06813-1)
Supplement: Supplementary file 1 — Additional file 1: Table S1. Summary of demography, baseline characteristics, and co-existing illness. [file 12879_2021_6813_MOESM1_ESM.docx]

**Additional Supplementary Table 1: Demography, Baseline characteristics, and Co-existing illness**

|  | **Overall**  **(N=738)** | **Control Group (N=476)** | **Tocilizumab Group (N=262)** | **P-value** |
| --- | --- | --- | --- | --- |
| **Age (Years), Mean (SD)** | 61.0 (14.72) | 61.9 (15.66) | 59.2 (12.65) | 0.0139^ |
| **Gender – Male, n (%)** | 527 (72.1) | 330 (69.8) | 197 (76.4) | 0.0577^^ |
| **Gender - Female, n (%)** | 204 (27.9) | 143 (30.2) | 61 (23.6) |  |
| **Weight (kg), Mean (SD)** | 81.0 (18.83) | 79.8 (18.37) | 83.2 (19.51) | 0.0255^ |
| **BMI, Mean (SD)** | 30.5 (8.31) | 30.3 (8.73) | 31.0 (7.48) | 0.0948^ |
| **GCS Baseline, Median (IQR)** | 15.0 (11.00, 15.00) | 15.0 (9.00, 15.00) | 15.0 (15.00, 15.00) | 0.0007^ |
| **APACHE II score, Median (IQR)** | 13.0 (8.00, 23.00) | 14.0 (8.00, 23.00) | 12.0 (7.00, 22.00) | 0.2545^ |
| **SOFA score, Median (IQR)** | 5.0 (3.00, 8.00) | 5.0 (3.00, 8.00) | 4.0 (3.00, 7.00) | 0.0343^ |
| **Systemic Corticosteroids use during ICU, n (%)** | 636 (88.0) | 395 (84.9) | 241 (93.4) | 0.0008^^ |
| **Serum creatinine mmol/l, Median (IQR)** | 90.0 (71.00, 138.00) | 94.0 (71.00, 148.00) | 87.0 (69.00, 125.00) | 0.0224^ |
| **eGFR Baseline (ml/min/1.73m^2), Median (IQR)** | 74.0 (43.00, 97.00) | 72.0 (39.00, 96.00) | 79.5 (53.00, 98.00) | 0.0063^ |
| **Acute Kidney Injury (AKI) within 24 hours of ICU admission, n (%)** | 212 (29.0) | 148 (31.3) | 64 (24.7) | 0.0606^^ |
| **MV during ICU stay within 24hr, n (%)** | 480 (65.8) | 303 (64.2) | 177 (68.6) | 0.2301^^ |
| **Lactic acid Baseline (mmol/l), Median (IQR)** | 1.9 (1.34, 2.65) | 1.8 (1.32, 2.60) | 1.9 (1.40, 2.75) | 0.3324^ |
| **Platelets count Baseline (10^9/l), Median (IQR)** | 252.0 (191.00, 326.00) | 245.0 (186.00, 329.00) | 256.0 (200.00, 323.00) | 0.2463^ |
| **Total WBC Baseline (10^9/l), Median (IQR)** | 10.0 (6.99, 14.00) | 10.3 (7.19, 13.95) | 9.3 (6.76, 14.10) | 0.2283^ |
| **International normalized ratio (INR), Median (IQR)** | 1.1 (1.04, 1.21) | 1.1 (1.04, 1.22) | 1.1 (1.04, 1.20) | 0.9807^ |
| **activated partial thromboplastin time (aPTT) Baseline (Seconds), Median (IQR)** | 29.9 (26.80, 33.30) | 29.9 (26.70, 33.70) | 29.7 (27.00, 32.60) | 0.2424^ |
| **Total bilirubin (umol/l), Median (IQR)** | 10.0 (7.00, 14.60) | 10.0 (7.00, 15.00) | 10.1 (7.30, 14.00) | 0.6736^ |
| **Alanine aminotransferase (ALT) Baseline U/L, Median (IQR)** | 38.0 (25.00, 66.00) | 37.0 (25.00, 68.00) | 40.5 (26.00, 64.00) | 0.6809^ |
| **Aspartate aminotransferase (AST) Baseline U/L, Median (IQR)** | 55.0 (35.00, 80.00) | 55.0 (33.00, 82.00) | 54.0 (36.00, 76.00) | 0.9043^ |
| **Albumin Baseline (gm/l), Median (IQR)** | 33.0 (29.60, 36.10) | 33.0 (29.00, 36.80) | 33.5 (30.00, 36.00) | 0.4690^ |
| **Blood urea nitrogen (BUN) Baseline mmol/l, Median (IQR)** | 7.5 (5.20, 14.30) | 8.0 (5.40, 15.20) | 6.9 (4.70, 12.00) | 0.0130^ |
| **CPK (U/l), Median (IQR)** | 200.0 (78.00, 510.00) | 189.0 (79.00, 467.00) | 233.0 (78.00, 563.00) | 0.3000^ |
| **CRP (mg/l) m Median (IQR)** | 155.0 (85.50, 223.00) | 143.0 (74.00, 208.00) | 167.0 (112.00, 248.00) | 0.0006^ |
| **Procalcitonin (ng/ml), Median (IQR)** | 0.3 (0.14, 1.35) | 0.3 (0.14, 1.45) | 0.3 (0.15, 1.05) | 0.7021^ |
| **Glu2 Baseline Within 24 hours of ICU admission (mmol/I), Median (IQR)** | 11.9 (8.00, 16.90) | 12.2 (8.40, 16.90) | 11.2 (7.60, 16.80) | 0.0934^ |
| **PaO2/FiO2 ratio within 24 hours of admission, Median (IQR)** | 82.5 (62.00, 126.00) | 87.8 (65.26, 143.30) | 75.5 (60.00, 108.35) | 0.0006^ |
| **PH within 24 hours of admission, Median (IQR)** | 7.4 (7.35, 7.46) | 7.4 (7.35, 7.46) | 7.4 (7.35, 7.46) | 0.8140^ |
| **Highest Heart Rate (HR) Baseline (beats per minute), Median (IQR)** | 103.0 (91.00, 115.50) | 103.0 (91.00, 116.00) | 104.0 (91.00, 115.00) | 0.9274^ |
| **Maximum body temperature Baseline (Celsius), Median (Q1,Q3)** | 37.4 (37.00, 38.20) | 37.4 (37.00, 38.10) | 37.5 (37.05, 38.40) | 0.1116^ |
| **VTE prophylaxis, n (%)** | 624 (86.2) | 400 (85.7) | 224 (87.2) | 0.5740^^ |
| **History of Hospitalization within 1 year, n (%)** | 85 (14.7) | 66 (20.5) | 19 (7.4) | <.0001^^ |
| **History of invasive procedure (Surgery) within 1 year, n (%)** | 85 (14.7) | 66 (20.5) | 19 (7.4) | <.0001^^ |
| **CRE History within 1 year, n (%)** | 6 (1.0) | 4 (1.2) | 2 (0.8) | 0.6315** |
| **Antibiotics Exposure in last 3 months, n (%)** | 85 (11.5) | 56 (16.9) | 29 (11.4) | 0.0588^^ |
| **Antibiotics Non-Exposure in last 3 months, n (%)** | 653 (88.5) | 420 (83.1) | 233 (88.6) |  |
| **Dyslipidemia (DLP)** | 165 (22.9) | 111 (24.0) | 54 (20.9) | 0.3433^^ |
| **Diabetes mellitus (DM)** | 440 (61.1) | 296 (64.1) | 144 (55.8) | 0.0293^^ |
| **Hypertension (HTN), n (%)** | 412 (57.2) | 272 (58.9) | 140 (54.3) | 0.2305^^ |
| **Acute Coronary Syndrome (ACS), n (%)** | 13 (1.8) | 10 (2.2) | 3 (1.2) | 0.3331** |
| **Asthma, n (%)** | 60 (8.4) | 42 (9.1) | 18 (7.0) | 0.3170^^ |
| **Atrial fibrillation (Afib.), n (%)** | 19 (2.6) | 11 (2.4) | 8 (3.1) | 0.5731^^ |
| **Chronic obstructive pulmonary disease (COPD), n (%)** | 15 (2.1) | 12 (2.6) | 3 (1.2) | 0.1924^^ |
| **Cancer (any type), n (%)** | 26 (3.7) | 18 (3.9) | 8 (3.1) | 0.5846^^ |
| **Chronic kidney disease (CKD)- (On Dialysis), n (%)** | 78 (10.7) | 61 (12.9) | 17 (6.6) | 0.0079^^ |
| **Coronary artery bypass grafting (CABG), n (%)** | 22 (3.1) | 14 (3.1) | 8 (3.1) | 0.9699^^ |
| **Heart failure (HF), n (%)** | 62 (8.6) | 46 (10.0) | 16 (6.2) | 0.0821^^ |
| **Hypothyroidism, n (%)** | 42 (5.8) | 29 (6.3) | 13 (5.0) | 0.4881^^ |
| **Ischemic heart disease (IHD), n (%)** | 62 (8.7) | 49 (10.7) | 13 (5.1) | 0.0105^^ |
| **Liver disease (any type), n (%)** | 15 (2.1) | 13 (2.8) | 2 (0.8) | 0.0642^^ |
| **Venous thromboembolism (VTE) , n (%)** | 11 (1.5) | 8 (1.7) | 3 (1.2) | 0.5463** |
| *T Test / ^ Wilcoxon rank sum test is used to calculate the P-value.  PS: Propensity score  ^^ Chi square/ ** Fisher’s Exact teat is used to calculate P-value. | | | | |
